# Comparison of Uterine Involution and the Resumption of Ovarian Cyclicity between Lame and Sound Holstein Cows

**DOI:** 10.3390/ani13233645

**Published:** 2023-11-25

**Authors:** Anastasia Praxitelous, Panagiotis D. Katsoulos, Angeliki Tsaousioti, Christos Brozos, Marion Schmicke, Constantin M. Boscos, Georgios Tsousis

**Affiliations:** 1 Clinic of Farm Animals, School of Veterinary Medicine, Faculty of Health Sciences, Aristotle University of Thessaloniki, 54627 Thessaloniki, Greece; praxitea@vet.auth.gr (A.P.); katsoulo@vet.auth.gr (P.D.K.); tsaoange@vet.auth.gr (A.T.); brozos@vet.auth.gr (C.B.); pboscos@vet.auth.gr (C.M.B.); 2 Clinic for Cattle, Veterinary Endocrinology and Laboratory Diagnostic, University of Veterinary Medicine Hannover, 30559 Hannover, Germany; marion.schmicke@tiho-hannover.de

**Keywords:** lameness, dairy cattle, uterine involution, ovarian resumption, energy status

## Abstract

**Simple Summary:**

Rapid and successful involution of the reproductive system is necessary for reproductive efficiency in dairy cows. This study aimed to examine the effect of lameness on uterine involution and the ovarian onset of cows with otherwise healthy puerperium. Retrospectively, the involvement of cows’ energy status and its interaction with lameness was assessed. Lame cows showed delayed involution of the reproductive tract (cervix and formerly pregnant uterine horn), lower ovulation rates, higher rates of atresia or cyst formation, and worse energy profiles regarding NEFA concentrations compared to sound cows. High NEFA concentrations affected these parameters separately and in relation to lameness, with the cows experiencing both conditions being most impacted regarding their reproductive physiology.

**Abstract:**

The aim of the study was to examine the effect of lameness and energy status on the involution of the uterus and the resumption of ovarian cyclicity in dairy cows. Lame (lameness score of four and the presence of hoof lesions, *n* = 22) and sound (normal gait and the absence of hoof lesions, *n* = 25) multiparous cows with healthy puerperium were enrolled simultaneously in the study and were monitored from day 10 antepartum (ap) to day 50 post-partum (pp). Ultrasonography of the cervix, the formerly gravid uterine horn and the ovarian structures was performed on d 8, 11, 14, 23, 30, and 42 pp. Blood sampling for progesterone, β-hydroxybutyrate (BHBA), and non-esterified fatty acids (NEFAs) was used to assess cyclicity and energy status. Lame compared to sound cows had higher NEFA concentrations on day 14 pp (0.54 ± 0.05 vs. 0.37 ± 0.05, respectively, *p* = 0.005), delayed involution of the cervix and the formerly pregnant uterine horn (*p* = 0.0003 and *p* = 0.02, respectively), lower ovulation rates within the experimental period (63.6% vs. 88%, respectively, *p* = 0.05), and higher rates of atresia or cyst formation on day 50 pp (36.4% vs. 12%, respectively, *p* = 0.05). Independently of lameness status, cows with high NEFA concentrations had lower ovulation rates within the experimental period (65.5% vs. 94.4%, *p* = 0.02), lower normal ovarian activity on day 50 pp (58.6% vs. 88.9%, *p* = 0.03), and higher rates of atresia or cyst formation on day 50 pp (34.5% vs. 5.6%, *p* = 0.02) compared to cows with optimal NEFA concentrations. Furthermore, an interaction between lameness and increased NEFA concentrations was observed regarding the ovulation rate within the experimental period and the percentage of atresia or cyst formation on day 50 pp. Sound cows with low NEFA levels had the lowest mean cervical diameter compared to cows with lameness (both with elevated and optimal NEFA concentrations, *p* = 0.009 and *p* = 0.002, respectively). Conclusively, lameness during puerperium negatively affected ovarian function and uterine involution. These effects were exacerbated (through interaction or cumulation) in relation to elevated NEFA concentrations.

## 1. Introduction

Modern dairy farming prioritizes welfare, as stress disrupts various physiological mechanisms that are essential for high-yielding cattle. Lameness is a significant stressor associated with deteriorated reproductive efficiency in affected animals [1,2]. It activates the hypothalamus–pituitary–adrenal (HPA) axis and can exert a direct effect on reproduction due to disturbances of the reproductive (hypothalamus–pituitary–gonadal) axis, among other indirect modes of action [3,4].

Uterine involution and the resumption of ovarian activity post-parturition are fundamental biological processes for the successful onset of the breeding period. Involution in dairy cows is hormone-dependent and has been investigated in a limited number of studies, primarily as an indicator of genital system health after calving. The reduction in the size of the uterus has been associated with the histological regeneration of the endometrium [5,6]. Although the biological importance of the precise day of uterus restoration is not self-evident and remains a matter of discussion, rapid and successful involution is a prerequisite for reproductive efficiency and has been associated with the resumption of ovarian activity after calving [7,8,9]. Parity, age, and Body Condition Score (BCS) at calving, as well as puerperal and metabolic diseases, have been studied regarding their effect on uterine involution [10,11,12]. Puerperal diseases and metabolic disorders are considered major suppressors of uterine involution [9,11] and affect endometrium remodeling [13,14]. In previous studies, lameness has been associated with an increased incidence of puerperal [15] and metabolic disorders [16,17]. However, to our knowledge, the effect of lameness on the involution of the cervix and the previously gravid horn has not yet been investigated.

Concurrently, follicular emergence is follicle-stimulating hormone (FSH) dependent and initiates on the fifth to seventh day after parturition. However, the first ovulation can be postponed until day 50 to 60 post-partum (pp), if the luteinizing hormone (LH) pulses or the preovulatory LH surge are uncoordinated or insufficient, leading to successive cycles of atresia or cyst formation. In the study by Garbarino et al. [18], cows with moderate or severe lameness showed 14% and 17% delayed onset of ovarian activity, based on measurements of progesterone concentrations during the first 35 days pp. Additionally, Melendez et al. found a lower ovulation rate in lame cows (5/10) compared to healthy ones (10/10) [19]. The direct effect of lameness on LH release was evident in the study by Morris et al. [3], where 30% of lame cows did not respond to a protocol for the synchronization of ovulation and another subgroup of cows responded, but did not ovulate, with them showing lower LH pulse frequencies. Moreover, both aforementioned groups had no detectable LH surge.

Another possible mechanism, by which lameness can impact reproductive efficiency, is the energy status of affected animals. Negative energy balance (NEB), defined across studies as body condition score reduction, body weight loss, reduced feed intake, or through the measurement of metabolic parameters, has been extensively related to impaired fertility and ovarian dysfunction [20,21,22,23]. NEB is responsible for disrupting LH release and impacts ovarian function, as reduced concentrations of insulin and insulin-like growth factor (IGF-1) can alter the responsiveness of the ovulatory follicle to LH and disrupt the fate of the first dominant follicle [24,25,26]. Additionally, there is evidence that lame cows frequently exhibit altered feeding behaviors [27,28] and low body condition [29,30]. Although both lameness and NEB affect the reproductive performance of dairy cows, and these conditions can interrelate, available literature investigating these interactions is scarce.

The present study aimed to profile the involution of the cervix and the previously gravid horn and the onset of the ovarian function of lame and non-lame cows with otherwise healthy puerperium, using B-mode ultrasonography and hormone analyses. Concurrently, the involvement of energy status in the aforementioned parameters and its interaction with lameness was investigated.

## 2. Materials and Methods

### 2.1. Ethical Statement

This study was approved by the Assembly of the Faculty of Veterinary Medicine, Aristotle University of Thessaloniki (69/30.06.2016). All procedures complied with the EU Directive 2010/63/CE.

### 2.2. Animals and Housing

The study was conducted on a single dairy farm with Holstein Friesian cows located in Analipsi, Thessaloniki, Greece, from July 2016 to December 2019. During the experimental period, the herd size was 200 cows, with an average milk production per cow per year of 10,542 kg. During the dry period, the cows were housed in a bedded pack barn on composted manure with an available outdoor loafing area. During the puerperal period and until day 50, the cows were housed in a bedded pack barn on straw. Both stalls were equipped with a 4 m feeding corridor with an automatic scraper system. For the rest of their milking period, the cows were housed indoors in free-stall barns with rubber floors, automatic scraper systems, and cubicles with mattresses. The animals were fed twice daily with total mixed rations formulated according to National Research Council (NRC) recommendations [31] and had free access to water.

### 2.3. Inclusion Criteria and Experimental Procedures

**Figure 1:**
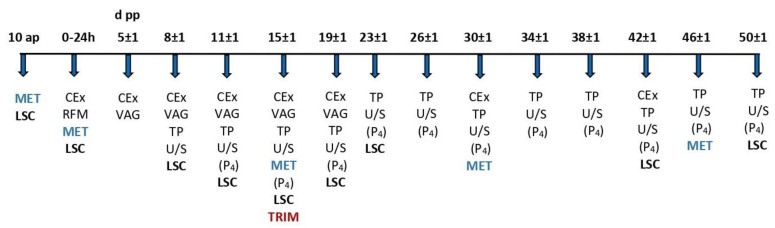
Experimental design—schematic representation. d pp = days post-partum, ap = antepartum, ΜΕΤ = blood sampling for metabolic parameters (NEFA and BHBA), LSC = lameness scoring, CEx = clinical examination, RFM = examination for retained fetal membranes, VAG = vaginoscopy, TP = transrectal palpation, U/S = transrectal ultrasonography, (P4) = blood sampling for the detection of progesterone concentration, and TRIM = conservative and therapeutic trimming.

Only multiparous cows were included in the study, as the uterus involution and ovarian resumption of primiparous cows have been found to follow diverse patterns [10,32]. All cows included in the study calved spontaneously (a score of 1 on the 5-point scale of calving ease [33]). Cases of dystocia, the birth of twins, and stillbirth were excluded from the study. The study cows were clinically examined on the day of calving and thereafter, as described in Figure 1. The clinical examination included measurement of body temperature, thoracic and abdominal auscultation and percussion, inspection and palpation of the udder and the teats, and examination of mammary gland secretion (the California mastitis test was performed in the parlor). Any cow that showed poor general condition during the study, pyrexia, and clinical signs of hypocalcemia, mastitis, abomasal displacement, or respiratory disease was excluded. In addition, cows with retained fetal membranes for more than 12 h or cows with abnormal uterine or vaginal discharge at any time point were removed from the study.

All cows were monitored from 10 days antepartum (ap, based on expected calving date) to 50 days pp. Any cow that met the above inclusion criteria and showed lameness up to day 15 pp, which was confirmed by lesion identification, was included in the study. At the same time, a homologous cow with normal gait and the absence of claw lesions was also selected. Three lame cows had to be removed from the study retrospectively due to missing samples. Finally, 22 lame and 25 control cows were included in the study.

The study involved the measurement of the structures of the genital tract of lame and non-lame cows during puerperium with the use of B-mode sonography. Additionally, blood samples were derived to detect progesterone concentration. B-hydroxybutyric acid (BHBA) and non-esterified fatty acids (NEFAs) were measured in blood serum to assess the cows’ energy status. All interventions, measurements, and samplings performed during the study are described below and are schematically presented in Figure 1:

- Day 10 ap: Blood sampling for the detection of BHBA and NEFA concentrations and lameness scoring (LSC).
- Calving day and within 24 h: Blood sampling for the detection of BHBA and NEFA concentrations, clinical examination, examination for retained fetal membranes, and lameness scoring.
- Day 5 ± 1 pp: Clinical examination, transrectal palpation, and vaginoscopy for the assessment of the genital system and uterine discharge.
- Day 8 ± 1 to 50 ± 1 pp: Clinical examination, lameness scoring, vaginoscopy, transrectal palpation, and ultrasound examination to record ovarian formations and the involution of the cervix and the formerly gravid uterine horn at the respective time points. In the case of the detection of an ovarian follicle larger than 10 mm, ultrasonography and blood sampling for the detection of progesterone concentration were repeated every 24–48 h until ovulation, atresia and regression, or cyst formation was confirmed. On day 15 ± 1, examination and trimming of all claws were performed on a standing chute, and lesions were recorded. All samplings, measurements, and treatments were performed by the first author.

### 2.4. Lameness Assessment and Recording

Cow gait was assessed while exiting the milking parlor using Sprecher’s five-point scale [34]. On day 15, eligible cows were led to the trimming chute. The control group (*n* = 25) was formed by cows that had normal gait (LSC = 1) in all measurements, both before and after their recruitment, and showed no lesions upon conservative trimming. Lame cows had a lameness score of 3 or 4 in at least 2 measurements until day 15. The lame group (*n* = 22) was formed by cows with sole or bulb ulcers, white line disease lesions, diffused sole/bulb hemorrhages with or without signs of laminitis (CHDL—claw horn disruption lesions, *n* = 11), and interdigital dermatitis with or without the presence of interdigital hyperplasia (ID—infectious diseases, *n* = 5) or with a combination of both conditions (*n* = 6). Cows with other lameness etiology (i.e., arthritis, acute injuries, or unspecified) were excluded from the study. Lesions were recorded, and appropriate therapy according to standard principles [35] was performed, including hoof blocks, bandages, anti-inflammatory drugs, and topical antibiotics in spray form.

### 2.5. Genital Examination and Ultrasonography

Vaginoscopy was performed after disinfection with a mild (5% concentration) iodine solution (Betadine^®^, Lavipharm, Athens, Greece) using a Götze vaginoscope and a light source. The lochia’s consistency, odor, quantity, and color were evaluated according to Sheldon’s definitions [36]. Until day 21, the finding of discharge that was initially reddish brown and gradually turned to greyish white, without an unpleasant odor or purulent admixtures, and no pyrexia present, was considered normal. After day 21, cows with no vaginal discharge or with the presence of clear mucus upon vaginoscopy were accepted. Transrectal palpation of the genital tract was used to evaluate the size of the cervix and the formerly gravid uterine horn, the horn symmetry, and the uterus contractility. These measurements were used for monitoring reasons and were not included in the statistical analysis.

The involution of the genital tract was estimated via transrectal B-mode ultrasonography with a 5 MHz linear transducer (Honda HS-101V; Honda Electronics Co., Ltd., Toyohashi, Japan). The diameter of the cervix was recorded approximately at the middle of its length, and the diameter of the formerly gravid uterine horn was approximately 2 cm cranially to the bifurcation. Cross-sectional images of the structures were stored, and the diameter was calculated as the mean of the maximum length and width. The procedure was performed thrice for every structure, and the mean value was used for statistical analysis. The ovaries were located afterward, and images of their structures (CL, follicles, and ovarian cysts) at their maximum size were stored. This procedure was also performed three times. All measurements of the anatomical structures were performed retrospectively with appropriate software (Inkscape^®^ version 1.0, New York, NY, USA).

### 2.6. Reproductive Definitions and Outcomes

To assess the resumption of ovarian activity, the formations of the ovaries along with the progesterone values of the same examination day were used, and the reproductive outcomes were defined accordingly:

- Ovulation: Identification of a dominant follicle >10 mm combined with P4 <1 ng/mL, the absence of the formation upon subsequent examination with ultrasonography 24–48 h later, and the development of a corpus luteum (CL) combined with an increase in P4 ≥1 ng/mL after 5 days [3,22,37].
- Atretic follicle (anoestrus, type II, according to Peter et al. [38]): Identification of a dominant follicle >10 mm combined with P4 <1 ng/mL, which did not ovulate and gradually regressed [37,38].
- Ovarian cyst (anoestrus, type III, according to Peter et al. [38]): Identification of an ovarian formation >25 mm in diameter for a period of at least ten days, without the presence of a CL or other functional formation in the same or bilateral ovary [22,38,39].
- Prolonged luteal phase (PLP, anoestrus, type IV, according to Peter et al. [38]): The presence of a CL which remained functional (P4 >1 ng/mL) for more than 20 days [22,38].

There were no cows with complete ovarian inactivity (anoestrus, type I, according to Peter et al. [38]).

At the end of the research period (day 50 pp) and based on the above definitions, each cow was classified into one of the following four categories:

- Cows with normal ovarian activity (cyclic) on day 50 pp: ovulation followed by the emergence of a CL and a new ovulation within an interval of ≤24 days (*n* = 33/47, 70.2%) [22,32]. In the case of a single ovulation in less than 24 days before the end of the experiment, its duration was extended for proper classification.
- Cows with an ovarian cyst on day 50 pp (*n* = 8/47, 17.0%).
- Cows with atresia on day 50 pp (*n* = 3/47, 6.4%).
- Cows with a prolonged luteal phase on day 50 pp (*n* = 3/47, 6.4%).

The following events regarding the entire experimental period were additionally recorded:

- Ovulation within the experimental period (*n* = 36/47, 76.6%).
- The day of the first ovulation.
- Formation of an ovarian cyst within the experimental period (*n* = 17/47, 36.2%).
- Prolonged luteal phase within the experimental period (*n* = 5/47, 10.6%).

### 2.7. Blood Sampling and Analytic Assays

Blood was always collected after morning milking and before feeding by coccygeal venipuncture into 10 mL vacuum polyethylene tubes without an anticoagulant (BD Vacutainer^®^, Becton, Dickinson and Company, Franklin Lakes, NJ, USA). The samples were stored in a common fridge and transferred with a cooler to be centrifuged (3000 rpm for 20 min) approx. 2–3 h after collection. Serum was transferred into Eppendorf-type tubes and stored at −20 °C until progesterone analysis and at −80 °C for the detection of metabolic parameters. Serum P4 concentration was determined by solid-phase RIA (gamma counter Wizard 1480, PerkinElmer, Turku, Finland) using a commercially available radioimmunoassay kit (IMMUNOTEC^®^, Prague, Czech Republic). The lower detection limit was 0.03 ng/mL. A progesterone concentration <1 ng/mL was considered indicative of the absence of active luteal tissue. BHBA and NEFA concentrations were determined by spectrophotometry (Pentra C400^®^, HORIBA ABX SAS, Montpellier, France). The cut-off for BHBA was set at 1.2 mmol/L [40]. The cut-off for NEFA varied and was set according to the findings of Ospina et al. [41] and Dubuc et al. [42], as follows:

- At calving: 0.9 mEq/L
- On day 15 pp: 0.6 mEq/L
- Antepartum and after day 15 pp: 0.4 mEq/L

The intra- and interassay coefficients of variation for all of the above analyses were <10%.

### 2.8. Statistical Analysis

The data were analyzed with the online platform SAS^®^ OnDemand for Academics (SAS Institute, Cary, NC, USA). The sample size was initially estimated for the continuous variables (BHBA, NEFA, and P4). A difference in the NEFA concentrations between the control and lame cows, similar to that found on day 15 pp (0.37 mEq/L vs. 0.54 mEq/L, with a standard deviation of 0.15), required a total sample size of 22 cows for the test power to exceed 0.80 and a total of 36 cows for the power to reach 0.95. However, in the present study, the test power did not exceed 0.80 for the binary variables. The normality of the distribution of continuous variables (BHBA and NEFA values and cervical and uterine horn diameter) was tested by the Shapiro–Wilk test. All parameters followed a normal distribution; thus, simple comparisons between independent groups were performed using the Student’s *t*-test, while repeated measures over time were analyzed using general linear mixed models. The proportions were compared using the χ^2^ test and Fisher’s exact test. Generalized linear mixed models were used to investigate the interactions between the presence of lameness and energy status in terms of reproductive outcomes and genital involution. Cows that exceeded the aforementioned cut-off limits (1.2 mmol/L at any time point for BHBA and 0.9, 0.6, and 0.4 mEq/L at calving, on day 15, and antepartum/after day 15, respectively, for NEFA) were categorized as BHBA+ and NEFA+. The statistical models included the fixed effects of the occurrence of lameness (yes or no), BHBA or NEFA values above the cut-off points (yes or no, in discrete models for the two variables), and the interaction between lameness and energy status. The data are presented as least-squares means, arithmetic means, or proportions, depending on the statistical procedure used. The significance level was set at 0.05.

## 3. Results

### 3.1. Energy Status of the Lame Cows

**Figure 2:**
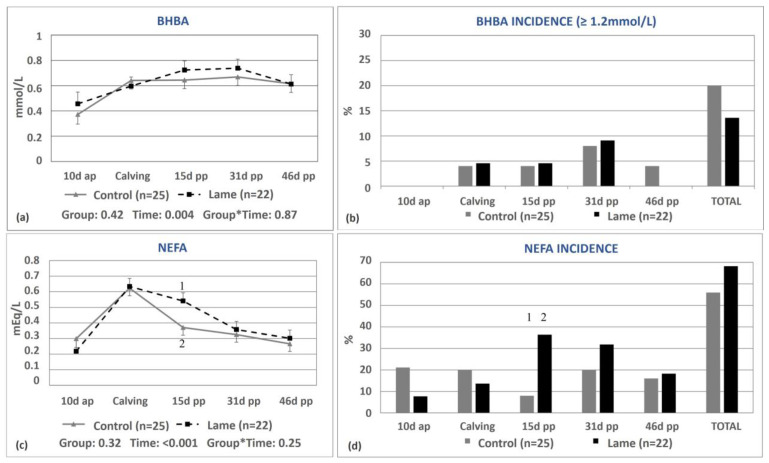
Least-squares means (±SE) and the incidence of elevated ß-hydroxybutyric acid (BHBA, (**a**,**b**)) and non-esterified fatty acids (NEFAs, (**c**,**d**)) in the control and lame cows at 10 days antepartum (ap), at calving, and at 15, 31, and 46 days post-partum (pp). NEFA incidence was calculated based on different thresholds depending on the day of measurement (at calving: 0.9 mEq/L, on day 15 pp: 0.6 mEq/L, antepartum, and after day 15 pp: 0.4 mEq/L). ^1, 2^ Different numbers denote significant differences (*p* ≤ 0.05). * denotes interaction.

No significant differences were observed between the lame group and the control group in terms of the mean β-hydroxybutyric acid concentrations (0.63 ± 0.03 vs. 0.59 ± 0.03 mmol/L, *p* = 0.42, respectively) or at any discrete time point (Figure 2a). During the study period, only 8 cows had BHBA concentrations greater than 1.2 mmol/L (3 out of 22, 13.6%, in the lame group and 5 out of 25, 20.0%, in the control group, *p* = 0.56, Figure 2b).

No significant differences were found between the lame and control cows regarding the overall concentration of non-esterified fatty acids (0.41 ± 0.02 vs. 0.38 ± 0.02 mEq/L, *p* = 0.32, respectively, Figure 2c). However, the lame cows experienced a slower decrease in NEFA concentration after calving compared to the controls, which resulted in a difference in NEFA concentrations between the two groups on day 15 pp (0.54 ± 0.05 vs. 0.37 ± 0.05 for lame and control cows, respectively, *p* = 0.005, Figure 2c). On the same day, the incidence of NEFA concentrations greater than the cut-off point was 36.4% in the lame cows versus 8% in the control cows (*p* = 0.02, Figure 2d). In all other measurements, no significant differences in NEFA concentration were observed between the two groups.

### 3.2. Uterine Involution in Relation to Lameness and Energy Status 

**Figure 3:**
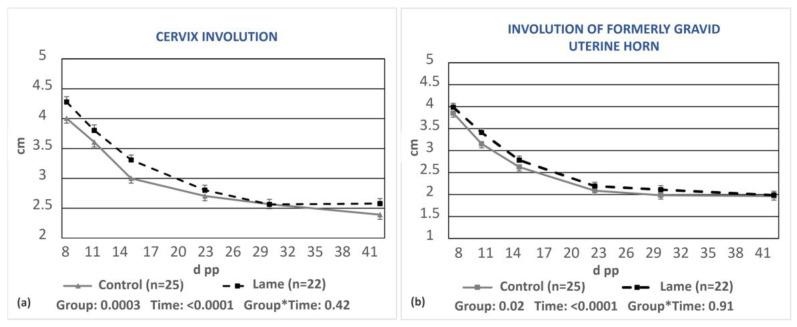
Least-squares means (±SE) of the diameters of the cervix (**a**) and the formerly gravid uterine horn (**b**) during puerperium in the control and lame cows. * denotes interaction.

A significant delay in the involution of both the cervix and the formerly gravid uterine horn was found in the lame group compared to the control group (*p* = 0.0003 and *p* = 0.02, respectively, Figure 3a,b).

**Figure 4:**
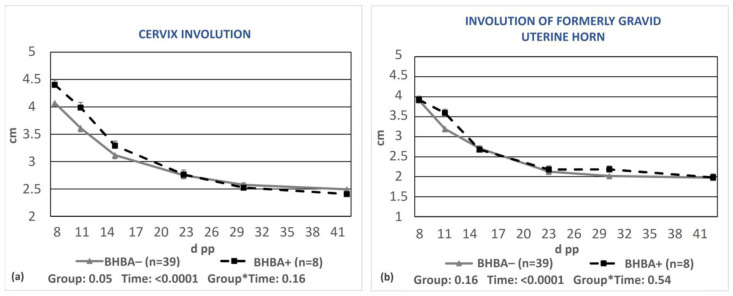
Least-squares means (±SE) of the diameters of the cervix (**a**) and the formerly gravid uterine horn (**b**) during puerperium in cows with BHBA ≥1.2 mmol/L (BHBA+) and cows with optimal BHBA concentrations (BHBA−). * denotes interaction.

A significant delay in cervix involution (*p* = 0.05, Figure 4a), but not regarding the formerly gravid horn (*p* = 0.16, Figure 4b), was observed in cows with BHBA concentrations greater than 1.2 mmol/L compared to cows with optimal BHBA values.

**Figure 5:**
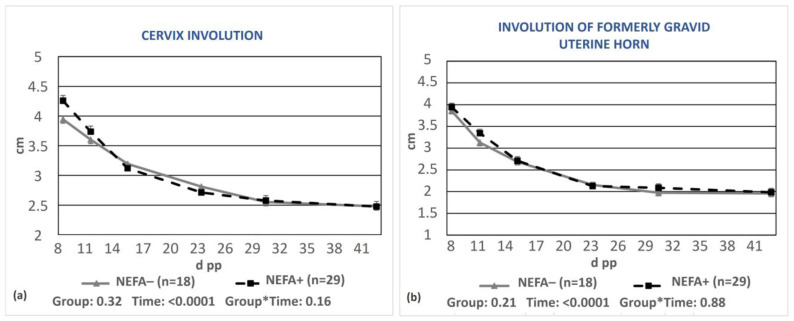
Least-squares means (±SE) of the diameters of the cervix (**a**) and the formerly gravid uterine horn (**b**) during puerperium in cows with NEFAs greater than the cut-off points (NEFA+) and cows with optimal NEFA concentrations (NEFA−). NEFA incidence was calculated based on different thresholds depending on the day of measurement (at calving: 0.9 mEq/L, on day 15 pp: 0.6 mEq/L, antepartum, and after day 15 pp: 0.4 mEq/L). * denotes interaction.

No significant differences were observed regarding the uterine involution of cows grouped according to blood NEFA concentrations (Figure 5a,b).

**Figure 6:**
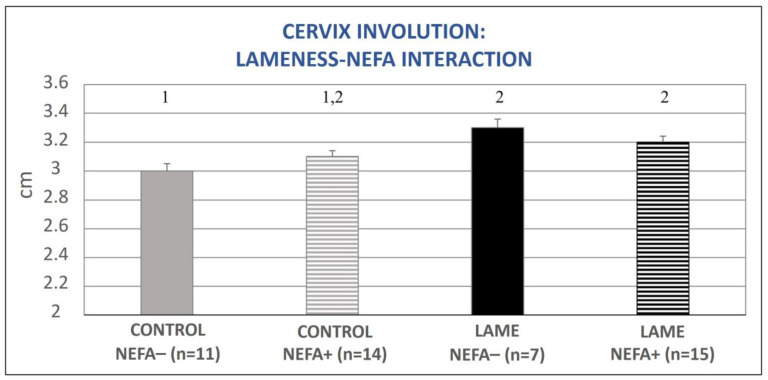
Lameness–NEFA interaction regarding cervix diameter during the entire puerperium period. NEFA incidence was calculated based on different thresholds depending on the day of measurement (at calving: 0.9 mEq/L, on day 15 pp: 0.6 mEq/L, antepartum, and after day 15 pp: 0.4 mEq/L). The results are presented as the least-squares mean (±SE). ^1, 2^ Different numbers denote statistical differences (*p* ≤ 0.05).

The mixed model analysis revealed no significant interaction between lameness and BHBA concentration regarding the involution of the cervix (*p* = 0.81) or the formerly gravid uterine horn (*p* = 0.90). There was also no interaction between lameness and NEFA concentration regarding the involution of the formerly gravid uterine horn (*p* = 0.26); however, a tendency regarding the involution of the cervix (*p* = 0.07, Figure 6) was found. Specifically, control cows with simultaneously low NEFA levels had the lowest mean cervical diameter and differed significantly from cows with lameness (both with elevated and optimal NEFA concentrations, *p* = 0.009 and *p* = 0.002, respectively, Figure 6).

### 3.3. Resumption of Ovarian Activity in Relation to Lameness and Energy Status

A significantly higher percentage of ovulation during puerperium was observed in the control group compared to the lame group (88.0% vs. 63.6%, *p* = 0.05, respectively, Table 1). Also, on day 50 pp, a significantly lower percentage of cysts or atretic follicles was observed in the control cows compared to the lame cows (12.0% vs. 36.4%, *p* = 0.05, respectively, Table 1). Finally, apart from prolonged luteal phases, the control cows showed numerically fewer ovarian activity disorders than the lame cows (Table 1).

Based on the measured blood BHBA concentrations, cows with concentrations lower than 1.2 mmol/L had numerically better results for most parameters compared to cows with elevated BHBA concentrations (Table 2); however, these differences were not significantly different.

Based on the measured blood NEFA concentrations, cows with optimal NEFA levels showed a significantly higher rate of ovulation during the experimental period (0 to 50 d pp) (94.4% vs. 65.5%, *p* = 0.02, respectively), a significantly higher rate of normal ovarian cyclicity on day 50 pp (88.9% vs. 58.6%, *p* = 0.03, respectively) and a lower proportion of ovarian disorders (ovarian cysts and atretic follicles) (5.6% vs. 34.5%, *p* = 0.02, respectively, Table 3) compared to cows with elevated NEFA concentrations.

**Figure 7:**
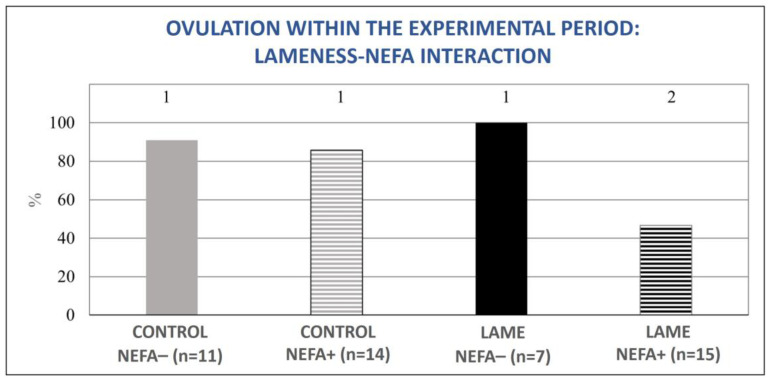
Lameness–NEFA interaction regarding the proportion of cows that ovulated during the experimental period (0–50 d pp). The results are presented as the least-squares mean (±SE). ^1, 2^ Different numbers denote statistical differences (*p* ≤ 0.05).

**Figure 8:**
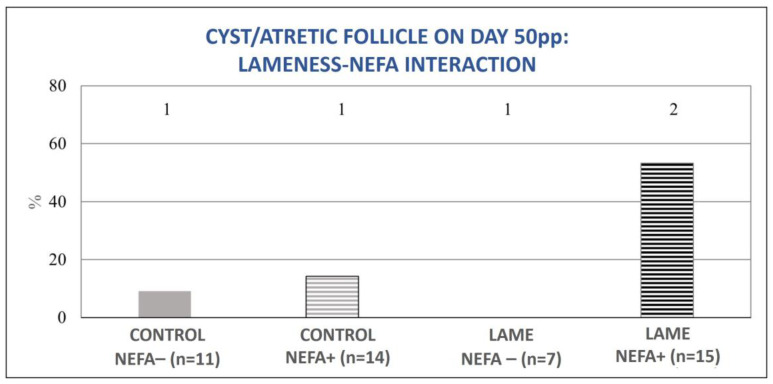
Lameness–NEFA interaction regarding the proportion of cows with an ovarian cyst or atretic follicle on day 50 pp. The results are presented as the least-squares mean (±SE). ^1, 2^ Different numbers denote significant differences (*p* ≤ 0.05).

The mixed model analysis revealed no significant interaction between lameness and high BHBA concentrations for the tested variables (ovulation during the experimental period (0–50 d pp), normal ovarian cyclicity on day 50 pp, and the presence of a cyst or an atretic follicle on day 50 pp). However, the interaction between lameness and high NEFA concentrations was significant for the variables of ovulation during the experimental period (0–50 d pp) and the presence of an ovarian cyst or atretic follicle on day 50 pp (*p* = 0.04, for both). In particular, cows with both lameness and elevated NEFA concentrations showed significant deterioration in the above parameters compared to all other subgroups (Figure 7 and Figure 8).

## 4. Discussion

A common difficulty encountered in studying the adverse effects of lameness on the reproductive efficiency of dairy cows is the diverse modes of action of the condition. In order to minimize as many confounding factors as possible, we paired lame with non-lame cows to avoid a seasonal/management effect. In addition, we did not use primiparous cows, as they follow different patterns regarding uterine involution and ovarian resumption compared to multiparous cows. Lastly, we excluded cows with any evidence of puerperal disorder, which could have exacerbated the negative effect of lameness, as lame cows are prone to metritis [15].

Lame cows showed delayed involution of the cervix and the formerly gravid uterine horn, while the effect of low energy status was not as evident. Only cows with high BHBA concentrations had a delay regarding cervical, but not uterine horn, regression. Additionally, increased NEFA concentrations did not have an independent effect on uterine involution. There was, however, an interaction with the concurrent presence of lameness regarding the involution of the cervix. Paiano et al. [14], studying the effect of different metabolic diseases on uterine involution, found a significant effect in cows with ketosis and in those with elevated NEFA concentrations antepartum. A critical difference compared to our methodology, however, was that cows with metritis or abnormal vaginal discharge had not been excluded in that study, thus a confounding effect might have occurred [9,11,43], especially taking into account that ketosis and uterine infections are associated [40,44]. The biological significance of the small differences in the recorded uterine involution is not self-evident and remains a matter of discussion. However, a delay in uterine involution has been associated with normal ovarian function and vice versa and could macroscopically reflect the cellular and biochemical changes occurring in the endometrium [6]. The slower uterine involution in lame cows compared to control cows was demonstrated for the first time in the present study and is a motivation for future research focusing on hormonal and immunoregulatory mechanisms.

In order to confirm normal ovarian function after parturition, it is important to follow up with cows beyond first ovulation, as many of them show irregular ovarian patterns [20]. The results of the present research suggest that, without accounting for energy status, lame cows had lower ovulation rates within the study period and more ovarian disorders (cysts/atretic follicles) on day 50 pp compared to non-lame cows, which is in accordance with the results of a previous study that demonstrated a higher incidence of cysts and atretic follicles between 28- and 37-days post-partum [45]. These outcomes are also supported by the research of Sood et al. [46], who studied the growth and developmental patterns of follicular waves in cyclic cows with (*n* = 10) and without (*n* = 6) lameness. They found that, although there were no differences between the two groups in the emergence of follicle waves, the lame cows showed delayed selection of the dominant follicle (8 vs. 3.6 days in the non-ovulatory wave and 5.6 versus 2.8 days in the ovulatory wave) and longer persistence of the dominant follicle (10.9 vs. 9.4 days) compared to the control cows. The results of other studies converge toward similar conclusions; Walsh et al. [23] showed that lame cows had a 1.8-fold higher risk of anovulation at 46–60 days pp, while Garbarino et al. [18], based on blood progesterone measurements, demonstrated that lame cows had a 3.5-fold higher risk of delayed ovarian resumption until day 60 pp compared to their healthy counterparts. Also, in agreement with the present study’s findings, Melendez et al. [47] observed that cows that developed lameness by day 30 pp were 2.6 times more likely to develop ovarian cysts before the first artificial insemination compared to healthy cows. Furthermore, in our study, within the experimental period, 12% of the control cows and 4.6% of the lame cows experienced prolonged luteal phases. For PLP to occur, ovarian activity has to be commenced, which was reduced in the lame cows compared to the control cows. Our results agree with Opsomer et al. [20], who reported a 13% PLP prevalence in healthy cows (vs. 55% in metritic cows, due to the endometrial inflammation that disrupts the production of uterine prostaglandins). However, PLP was the only ovarian aberration for those cows that did ovulate within the experimental period, as no cow developed a cyst or an atretic follicle after ovulation had occurred and within the frame of this study. From these findings, we conclude that the incidence of first ovulation is an inadequate parameter to classify ovarian resumption during puerperium if accounted for alone; however, it can be very useful in relation to other indices.

The present study is one of the very few that have studied the energy status of lame cows and the possible interactions regarding their effect on the reproductive system. As mentioned above, there were only a small number of cows with elevated BHBA (*n*= 8/47), which is in alignment with the results of a previous study (*n*= 7/79) by our working group [45]. This fact resulted in difficulty in detecting significant differences and safely interpreting our data, although numerical differences existed between the BHBA− and BHBA+ groups regarding the indexes of ovarian resumption. However, the BHBA concentrations did not differ between the lame and control cows, neither statistically nor numerically. In the study by Garbarino et al. [18], ketosis was a risk factor for delayed ovarian resumption (2.76 times greater compared to cows with no ketosis); however, no interaction was evident regarding lameness, which functioned as an independent risk factor for anovulation. In the study by Sun et al. [48], the BHBA concentrations did not differ between the healthy cows and cows with LSC of 2 or 3, yet they were found to be significantly lower in cows with severe lameness, which was attributed by the authors to possible lower milk production by lame cows which permits them from meeting their energy requirements regardless of the lower feed intake. In the study by Daros et al. [15], lameness during the dry period was not directly associated with subclinical ketosis after calving. However, cows with lameness consumed reduced amounts of dry matter in the dry period, which could act as a predisposing factor for ketosis. Also, in a recent study, BHBA values were determined in cows at the time of hoof trimming. The number of cows with lesion identification and concurrent lameness with LSC > 3 was 19, of which only 2 suffered from ketosis [49]. In contrast, Calderon and Cook [17] found an association between elevated concentrations of BHBA 5–13 days post-partum and the incidence of moderate or severe lameness approximately 2 weeks before calving. The differences between the research protocols regarding chronicity, degree, and type of lameness could explain the above discrepancies. Additionally, it should be noted that cows with poor clinical conditions, post-partum diseases, or even severe lameness were excluded from the present study, and thus, the cows that formed the two groups showed a better energy status compared to studies with less strict exclusion criteria.

The NEFA cut-offs in the present study were set considering the risk of anovulation after calving, as described by Dubuc et al. [42]. Lame cows had both higher NEFA levels and higher elevated NEFA incidence compared to the non-lame group on day 15 pp. Elevated NEFA levels in lame cows are reasonably expected, as lameness is related to lower feed intake, BCS loss, and deeper negative energy balance, and are in accordance with the results of Kougioumtzis [50] who found a positive correlation between elevated NEFA levels and lameness incidence in various lactational stages. Conversely, in the studies of Melendez et al. [19] and Sun et al. [48], NEFA concentrations in lame cows were lower than those of healthy cows. In the first study [19], the authors attributed this finding to the chronicity of lameness which could result in a chronic catabolic state. In the study of Sood et al. [46], the NEFA concentrations between lame and control cows were similar, probably due to the moderate and fast recovering type of lesions included in the study.

The elevated NEFA concentrations within two weeks of calving had a pronounced negative effect on ovarian activity during puerperium, especially regarding the ovulation rate within the experimental period, restoration of normal ovarian activity on day 50 pp, and ovarian disturbances (the presence of cysts or atretic follicles). These results agree with Paiano et al. [14], who found that cows with elevated NEFA levels one week before calving had a 15-day delay in ovarian resumption compared to cows with optimal NEFA levels. Furthermore, examining the interaction between lameness and high NEFA concentrations, the authors also noticed that in cows with elevated NEFA levels and concurrent lameness, the ovulation rate was only 46.7% during the study, whereas the presence of cysts or atretic follicles at day 50 pp was 53.3%, rates that differed significantly from all other groups. Actually, based on our results regarding the onset of ovarian activity, the separate negative effect of lameness disappears and that of elevated NEFA weakens when the interaction term is implemented. According to our results, the co-occurrence of lameness and negative energy balance during the early (day 15) post-partum period escalates the risk of ovarian disorders in dairy cows. This finding indicates a stronger effect of the negative energy balance when it occurs closer to calving; as in a previous study of our working group, low energy status later in puerperium did not have the same impact as the aforementioned factor [45]. However, this important finding has to be verified by studies with larger sample sizes and increased power.

## 5. Conclusions

Lameness impaired uterine involution and ovarian resumption in cows during the puerperal period. Lame cows had higher NEFA concentrations on day 15 pp than the controls. Elevated NEFA levels independently and cumulatively affected the resumption of ovarian activity in the cows. Cows with lameness and concurrent elevated NEFA levels were most affected regarding their reproductive physiology. Based on the results of the present study, stakeholders and veterinarians should strive to prevent the lameness of dairy cattle during the transition period to minimize its negative effects on future reproduction.

## Figures and Tables

**Table 1:** Ovarian resumption and ovarian disorders during puerperium in the control and lame cows.

	Group	
Variable	Control	Lame
n	25	22
Ovulation between days 0 and 50 pp (%)	88.0 ^1^	63.6 ^2^
Day of 1st ovulation (mean ± SE)	27.3 ± 2.8	29.3 ± 3.5
Ovarian cyst between days 0 and 50 pp (%)	36.0	36.4
Prolonged luteal phase between days 0 and 50 pp (%)	12.0	4.6
Normal ovarian cyclicity on day 50 pp (%)	80.0	59.1
Ovarian cyst on day 50 pp (%)	12.0	22.7
Atretic follicle on day 50 pp (%)	0.0	13.6
Ovarian cyst or atretic follicle on day 50 pp (%)	12.0 ^1^	36.4 ^2^
Prolonged luteal phase on day 50 pp (%)	8.0	4.6

SΕ: standard error. pp: post-partum ^1, 2^ Different numbers denote significant differences between the groups (*p* ≤ 0.05).

**Table 2:** Ovarian resumption and ovarian disorders during puerperium in cows with elevated (BHBA+) or optimal (BHBA−) ß-hydroxybutyric acid concentrations in their blood.

	Group	
Variable	BHBA−	BHBA+
n	39	8
Ovulation between days 0 and 50 pp (%)	79.5	62.5
Day of 1st ovulation (mean ± SE)	28.3 ± 2.4	26.8 ± 5.6
Ovarian cyst between days 0 and 50 pp (%)	33.3	50.0
Prolonged luteal phase between days 0 and 50 pp (%)	7.7	12.5
Normal ovarian cyclicity on day 50 pp (%)	74.4	50.0
Ovarian cyst on day 50 pp (%)	18.0	12.5
Atretic follicle on day 50 pp (%)	2.6	25.0
Ovarian cyst or atretic follicle on day 50 pp (%)	20.5	37.5
Prolonged luteal phase on day 50 pp (%)	5.1	12.5

SΕ: standard error. pp: post-partum.

**Table 3:** Ovarian resumption and ovarian disorders during puerperium in cows with elevated (NEFA+) or optimal (NEFA−) non-esterified fatty acid concentrations in their blood. NEFA incidence was calculated based on different thresholds depending on the day of measurement (at calving: 0.9 mEq/L, on day 15 pp: 0.6 mEq/L, antepartum, and after day 15 pp: 0.4 mEq/L).

	Group	
Variable	NEFA−	NEFA+
n	18	29
Ovulation between days 0 and 50 pp (%)	94.4 ^1^	65.5 ^2^
Day of 1st ovulation (mean ± SE)	28.6 ± 3.3	27.5 ± 2.9
Ovarian cyst between days 0 and 50 pp (%)	27.8	41.4
Prolonged luteal phase between days 0 and 50 pp (%)	11.1	6.7
Normal ovarian cyclicity on day 50 pp (%)	88.9 ^1^	58.6 ^2^
Ovarian cyst on day 50 pp (%)	5.6	24.1
Atretic follicle on day 50 pp (%)	0	10.3
Ovarian cyst or atretic follicle on day 50 pp (%)	5.6 ^1^	34.5 ^2^
Prolonged luteal phase on day 50 pp (%)	5.6	6.9

SΕ: standard error. pp: post-partum ^1, 2^ Different numbers denote significant differences between the groups (*p* ≤ 0.05).

## Data Availability

Data are contained within the article. Data are available from the authors upon request.
